# Sensory-Motor Modulations of EEG Event-Related Potentials Reflect Walking-Related Macro-Affordances

**DOI:** 10.3390/brainsci11111506

**Published:** 2021-11-13

**Authors:** Annalisa Tosoni, Emanuele Cosimo Altomare, Marcella Brunetti, Pierpaolo Croce, Filippo Zappasodi, Giorgia Committeri

**Affiliations:** Department of Neuroscience, Imaging and Clinical Sciences, Gabriele d’Annunzio University, Via Luigi Polacchi 11, 66100 Chieti, Italy; emanuele.altomare@gmail.com (E.C.A.); marcella.brunetti@unich.it (M.B.); pierpaolo.croce@unich.it (P.C.); f.zappasodi@unich.it (F.Z.); giorgia.committeri@unich.it (G.C.)

**Keywords:** affordances, walking locomotion, framing distance, event-related potentials, spatial environment

## Abstract

One fundamental principle of the brain functional organization is the elaboration of sensory information for the specification of action plans that are most appropriate for interaction with the environment. Using an incidental go/no-go priming paradigm, we have previously shown a facilitation effect for the execution of a walking-related action in response to far vs. near objects/locations in the extrapersonal space, and this effect has been called “macro-affordance” to reflect the role of locomotion in the coverage of extrapersonal distance. Here, we investigated the neurophysiological underpinnings of such an effect by recording scalp electroencephalography (EEG) from 30 human participants during the same paradigm. The results of a whole-brain analysis indicated a significant modulation of the event-related potentials (ERPs) both during prime and target stimulus presentation. Specifically, consistent with a mechanism of action anticipation and automatic activation of affordances, a stronger ERP was observed in response to prime images framing the environment from a far vs. near distance, and this modulation was localized in dorso-medial motor regions. In addition, an inversion of polarity for far vs. near conditions was observed during the subsequent target period in dorso-medial parietal regions associated with spatially directed foot-related actions. These findings were interpreted within the framework of embodied models of brain functioning as arising from a mechanism of motor-anticipation and subsequent prediction error which was guided by the preferential affordance relationship between the distant large-scale environment and locomotion. More in general, our findings reveal a sensory-motor mechanism for the processing of walking-related environmental affordances.

## 1. Introduction

Animals’ survival in a highly challenging environment offering multiple possibilities of actions requires constant adaptation and flexibility of behavior. One strategy to adapt in such a multidimensional environment and select the best course of action at short notice is to specify the parameters of all possible actions in advance and maintain these representations in working memory until one is selected for overt execution.

This pragmatic and action-oriented view of the brain functional organization is conceptually formalized in the model of the “affordance competition hypothesis” [1,2]. According to this model, the brain functional organization, and specifically, the activity of sensory-motor regions in the dorsal fronto-parietal network, would be primarily dedicated to the transformation of information collected by the sensory systems into representations of potential actions. In particular, regions in the dorsal fronto-parietal cortex known to contain a rich mixture of sensory, motor and cognitive properties (i.e., sensory-motor transformation and visually guided action) would directly participate in the process of extraction of the features from the environment that afford action. Therefore, within this view, the primary role of these regions would be to provide a set of multiple representations of adaptive interactions with the environment.

This theorization of a neural architecture organized to satisfy the need of interactive behavior closely follows the original Gibsonian notion of affordance as action opportunities offered by the environment to perceiving organisms with reference to their individual’s action capabilities [3,4]. At the psychological–cognitive level, the model that best recapitulates the Gibsonian idea of (environmental) affordances is the embodied cognition view, a theoretical framework which posits that perceptual processes, including perception of the spatial environment, are strongly influenced by the observer’s body state and implicit representation of action [5,6].

On this basis, an hypothesis was formulated in our previous work [7] about a potential affordance relationship between the spatial features of the extrapersonal environment and locomotion, a behavior that is essential for survival and ubiquitous among mobile organisms. In particular, since locomotion appears as the privileged form of action to cover distance and retrieve information contained in the distant extrapersonal/environmental space, a facilitation effect was hypothesized for a walking-related action to distant vs. near objects/locations in the extrapersonal space. Behaviorally, this facilitation effect was expected to take the form of a specific response time (RTs) advantage for the execution of a footstep action, as proxy of locomotion, to pictures framing an object/environment from a far/panoramic vs. a near/restricted perspective. In our previous study, this hypothesis was tested using an incidental go/no-go priming paradigm requiring the execution of a walking-related action (i.e., a footstep ahead) in response to repeated presentations of pictures of an environmental layout framed from a far/panoramic vs. near/restricted view with respect to the observer [7]. Pictures were presented in pairs (prime and target) and the footstep action was executed at the onset of the target picture on the basis of the perceptual match with the prime picture.

Consistently with our predictions, a facilitation effect was observed for the execution of a walking-related action in response to distant (vs. near) objects/locations in the extrapersonal space, and this effect has been referred to as “macro-affordance” to reflect the parallelism with the well-known effect of “micro-affordance” observed during the execution of functionally appropriate hand-related actions towards manipulable objects positioned in the peripersonal space [7]. With respect to the model of the “affordance competition hypothesis”, in particular, one can hypothesize that prime pictures of a far/panoramic environment facilitate the coding of an environmental affordance enhancing the planning/execution of motor program potentially appropriate to cover distance and retrieve information from the far extrapersonal space, i.e., locomotion. This prime-related facilitation effect for locomotion would then be manifested and observable during presentation of the target stimulus.

Additional results from a recently published TMS study conducted in our lab [8] have indicated that this facilitation effect was abolished following TMS stimulation of a foot-related region located in the dorsal precuneate cortex [9,10,11] and included in the classic reaching-related dorsal stream for “perception for action” [12,13] as well as in the more recently described parieto-medial temporal pathways for spatial cognition and navigation [14]. These findings support the hypothesis of an early involvement of regions in the dorso-medial parietal cortex and connected frontal regions in the early processing/priming of environmental distance for the planning of whole-body actions, i.e., locomotion.

Nevertheless, since the TMS stimulation was administered off-line before the beginning of the experiment, no information could be extracted about the timing of the sensory-motor system involvement in the “macro-affordance” effect. To verify this hypothesis and investigate the whole-brain correlates of the macro-affordance effect, here, we used electroencephalography (EEG) during the same incidental priming paradigm employed in our previous studies [7,8]. Specifically, we investigated whether the walking-related “macro-affordance” effect was associated with a significant modulation of the neurophysiological activity during processing of the prime and target stimulus and the timing of these modulations. To this aim, a data-driven approach was employed to determine whether the EEG event-related potentials (ERPs) recorded during the prime-target interval were modulated by the framing distance of the environmental layout (i.e., far/panoramic vs. near/restricted view).

## 2. Materials and Methods

### 2.1. Participants and Testing

EEG study: a sample of 30 right-footed healthy participants (mean age: 22.2; 21 males) with no history of psychiatric or neurological disorder was recruited and participated in the EEG study.

Stimulus rating: a sample of 95 participants (mean age: 22.6) was enrolled for an explicit stimulus rating conducted through an online questionnaire aimed at evaluating the locomotion-affording properties of the stimulation set. The group of 95 participants enrolled for stimulus rating was independent and non-overlapping with the EEG group.

All participants had normal or corrected-to-normal vision and were naïve as to the purposes of the experiments.

The study was conducted in accordance with the ethical standards of the 1964 Declaration of Helsinki and was approved by the Ethics Committee of G. d’Annunzio University of Chieti, Italy.

### 2.2. EEG Study Stimuli and Apparatus

The experimental set-up was constituted by a 42″ wide screen and a foot-pedal response system (BrainTrends ltd., Rome, Italy) positioned on the floor at a distance of 57 cm from the screen. During the experiment, pictures were projected on the screen to cover about 70° of the visual angle. Stimulus presentation was controlled by the E-Prime software (Psychology Software Tools Inc., Pittsburgh, PA, USA) while foot-related responses were recorded through a foot-pedal response system connected with the E-Prime software.

**Figure 1:**
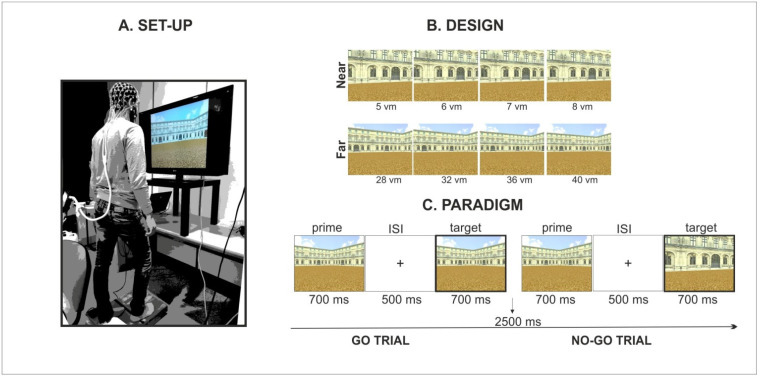
Experimental set up and behavioral paradigm. (**A**). Experimental set-up of wide-field stimulation and foot-pedal response system. (**B**). Experimental design including stimulation conditions of far and near extrapersonal distance (distance is expressed in virtual meters, vm). (**C**). Go/no-go incidental priming paradigm.

Foot-related responses were provided by releasing the foot pedal with the heel of the right foot, executing a footstep ahead at the onset of the target picture and then getting back to the starting position (Figure 1A). Participants were clearly instructed not only to move the leg for a step length but also the entire body ahead as if they were about to start walking. As a result, during the footstep action, the body weight was shifted from the non-responding left foot to the responding right foot, and this could be correctly executed without falling off the platform or running into the equipment. As discussed in our previous work [7], the footstep action was considered a proxy for walking because of their shared basic postural, muscular and kinematic elements.

Stimuli included a selection of pictures from a virtual reality environment, created by means of a 3D modelling software (3D Studio Max 4.2, Autodesk, Discreet), representing a square arena of a three-winged palace without any object on the floor. Specifically, pictures were created on the basis of the stimulation set employed in our original study [7] by simply removing the objects (i.e., beach umbrella and fountain) from the virtual environment [8]. Therefore, pictures only included the environmental layout defined by a three-winged palace on a square arena in which the framing distance was manipulated across two levels (far/panoramic vs. near/restricted view). As illustrated in Figure 1B, distance was manipulated across trials by framing the environment from different (extrapersonal) distances from the observer (from 5 to 8 virtual meters for the near condition and from 28 to 40 virtual meters for the far condition).

Pictures were rendered by moving the camera at different extrapersonal distances along a vector connecting the camera with the three-winged palace in the arena. The conditions of near and far distance were presented in randomized order. Distances were expressed in virtual meters, which were previously estimated as approximately doubled with respect to real distances in the current environment [15], and were selected with particular reference to the Grüsser’s distinction between a near-distant and a far-distant extrapersonal action space [16]. In particular, near and far locations were selected to fall very far from the proposed ~8 m (i.e., 16 virtual) boundary defining a near- vs. far-distant extrapersonal action space [15,16].

### 2.3. EEG Study Experimental Procedure

As illustrated in Figure 1C, the experimental design was based on an incidental go/no-go priming paradigm involving the presentation of picture pairs (i.e., prime and target image) and requiring subjects to execute a footstep action only when the target picture was perceptually identical to the prime picture (i.e., go trials). Subjects were instructed to refrain the response (i.e., no-go trials) when the pictures were different (note that prime and target pictures were selected from different distance’ categories, i.e., near/far and vice versa, thus resulting in a very easy discrimination).

Each picture was presented for 700 ms with an inter-stimulus-interval (ISI) of 500 ms. An inter-trial-interval (ITI) of 2500 ms was presented between every trial (pictures pair). Each subject completed 6 experimental blocks including 32 go trials (target picture equal to prime picture) and 8 no-go trials (target picture different from prime picture). The response was provided at the onset of the target picture and the reaction times associated with the foot pedal release were recorded on each trial. Notably, the no-go trials were exclusively included in the design to control participants’ attention to the relevant dimension and to ensure that the footstep response was not automatically executed at the onset of the target image. Before the beginning of the experiment, a brief training was performed on each participant in order to ensure that the task instructions were correctly understood and that the footstep action was accurately executed (i.e., see above for kinematic description).

### 2.4. EEG Study Behavioral Data Analysis

The mean accuracy performance in the go/no-go task, defined as the percentage of correctly executed footstep actions (i.e., go trials), and the mean response time, defined as the mean release time for the footstep action from the onset of the target picture, were calculated for each subject and distance condition and compared though paired *t*-tests (Student T, two-tails, type I).

The mean rating scores for the three dimensions of viewer–environment interaction were computed for each distance condition and compared through a within-subject repeated-measures ANOVA with distance (near, far) and condition of viewer–environment interaction (spatial exploration, reaching of object/location, passive admiration) as factors. The Newman–Keuls post-hoc test was used for statistical comparisons between the mean scores in the different conditions.

### 2.5. EEG Recording

EEG data were recorded by means of a 128-electrode net (version 1.1, Electrical Geodesic), placed according to an augmented 10–20 International System. The skin/electrode impedance was measured before each recording and kept below 50 kΩ. Simultaneous electrocardiographic activity was acquired. The EEG data were sampled at 250 Hz and processed offline.

### 2.6. EEG Data Analysis

Data preprocessing was partially performed by means of EEGLAB toolbox [17]. EEG data were backward–forward filtered between 1 and 40 Hz (second order Butterworth filter). Saturated or corrupted EEG epochs were rejected by visual inspection. A semiautomatic Independent Component Analysis-based procedure [18,19] was applied to identify and to remove cardiac and/or ocular artifacts, as well as activity coming from contraction of head muscles during movement or instrumental noise. For artefactual EEG identification, a set of criteria based on scalp topography and time-frequency features was used, as well as a neural network classifier, previously described [18,19,20]. EEG signals were transformed to the common average reference.

Data were segmented in trials from 1500 ms before to 1500 ms after the onset of the target picture. At least 40 cleaned epochs (N = 84 on average) were considered for each condition (near, far) for each subject. The baseline was chosen in the period of 300 ms preceding the onset of the prime picture (i.e., from −1500 and −1200 ms).

To examine ERPs modulation by execution of the footstep action in response to pictures framing the environment from a far/panoramic vs. a near/restricted perspective (i.e., far vs. near extrapersonal distance), we compared the ERP signals associated with the two conditions in a temporal window of 2200 ms spanning from −1200 to 1000 ms, i.e., from the onset of the prime picture to approximately the onset of the movement.

A Multivariate Pattern Analysis (MVPA) approach was used. MVPA aims to “decode” the brain activity predicting a model from the data (encoding approaches do the reverse, predicting data from a model, [21]). This approach differs from classical approaches in the fact that it takes into account at once multiple variables (channels and time), instead of considering them as independent (as in classical ERP component analysis). The goal of the decoding analysis is to test whether we can predict if the participant was responding to a specific task condition based on his/her patterns of brain activation, i.e., on the spatio-temporal structure of single-trial EEG. If EEG activity of single trial can successfully predict whether that trial corresponds to a near or a far extrapersonal condition, we can conclude that some information relevant to the experimental manipulation exists in the EEG data.

Here, MVPA was performed using the MVPA-Light MATLAB toolbox [22]. In particular, a Linear Discriminant Analysis (LDA) classifier was trained for each time point of the single trial EEG (time generalization analysis) in order to predict the two conditions (near vs. far). Such analysis provides a time course of accuracy. Intervals of values of accuracy above the chance threshold can be considered as intervals in which the model can decode the task. In our case, with two conditions, 50% accuracy is the guessing rate. If the classifier performs with an accuracy higher than that expected by chance, it provides evidence that the classifier can successfully generalize the learned associations to labelling new brain response patterns. As EEG data are inherently noisy and the separation could not be necessarily perfect [23], we choose a chance threshold value of 60%, as previously done.

For a classification with two classes, LDA calculates a decision value dval for a test EEG dataset x (corresponding to a channel X time matrix) as:

(1) $$\mathrm{dval}={\;w}^{T}x\;+\;b$$

where w is the weight vector or normal to the hyperplane specifying the linear combination of features, and b is the threshold/bias term [24]. If dval > 0, x is assigned to the first class and if dval < 0, it is assigned to the second class. If the classes near and far are respectively encoded as +1 and −1, this can be concisely expressed as:

(2) $$\mathrm{predicted}\;\mathrm{class}=\mathrm{sign}\left(w^{T}x\;+\;b\right)$$

where sign: ℝ → {−1, +1} is the sign function.

The LDA was trained in a Cross Validation (CV) framework [25]. Single EEG trials were divided in folds and the model was trained on all data except one fold in an iterative manner. The out-of-sample performance (i.e., generalization) is assessed based on the remaining fold and averaged across iterations. If the number of folds equals the number of EEG trials (one fold per trial: in our case all trials belonging to one subject) the procedure is defined as leave-one-out CV [26]. In this work, a leave-one-out CV was performed to train the LDA classifier ensuring generalization.

To assess if accuracy is statistically different from the chance accuracy level (60% in our case), a non-parametric permutation test was performed. In particular, the training and test procedure was repeated 100,000 times, shuffling the task labels across trials. An empirical density probability distribution was generated for each time point from −1200 to 1000 ms and accuracy values higher than the 99th percentile of the distributions were considered as significantly different from chance with a *p* < 0.01. In this way, a time course of accuracy was obtained. Whenever a time interval in which a significant difference near vs. far was evidenced, the mean values in this interval of each EEG channel were evaluated and a paired *t*-test was applied to evidence the scalp topography corresponding to the maximal near vs. far difference.

To determine the underlying cortical sources of evidenced scalp topography differences, we applied eLORETA [27] to the grand average of scalp potential distribution. The volume conductor model was given by a boundary element method (BEM) [28] of a template brain (http://www.bic.mni.mcgill.ca/ServicesAtlases/Colin27, Accessed on 10 February 2021) and the source space was modeled by a Cartesian 3D grid bounded by the template anatomy with 5113 voxels. Visualization of cerebral sources was performed using the connectome workbench (https://www.humanconnectome.org/software/connectome-workbench, Accessed on 10 February 2021).

### 2.7. Stimulus Rating

In addition to the go/no-go task, an explicit rating of the locomotion-related affording properties of the stimulation set was also collected on an independent and non-overlapping sample of participants with respect to the EEG sample. The rating was conducted through an online questionnaire administered via the Qualtrics commercial platform.

During the questionnaire, participants were shown with a stimulus exemplar from the near and far condition, and for each picture, a rating score was collected on the following affording conditions of viewer–environment interaction (i.e., affordance): i. going for a walk and exploring the environment (spatial exploration); ii. reaching of a specific object or spatial location (reaching of object/location): iii. passively admiring the environment (passive admiration). Responses were provided on a 7-point Likert scale. Therefore, a rating score for each of the three conditions of viewer–environment interaction was collected for each participant (N = 95) and stimulus picture (N = 2).

## 3. Results

### 3.1. EEG Behavioral Results

At the behavioral level, we firstly examined the accuracy performance in the go/no-go task (i.e., prime-target perceptual match). Consistently with our previous findings, analysis of the percentage of correct responses (i.e., footstep execution during go trials) indicated an overall high accuracy (mean accuracy across far and near conditions = 98%) with no significant difference between the conditions (paired *t*-test, *p* = 0.8).

**Figure 2:**
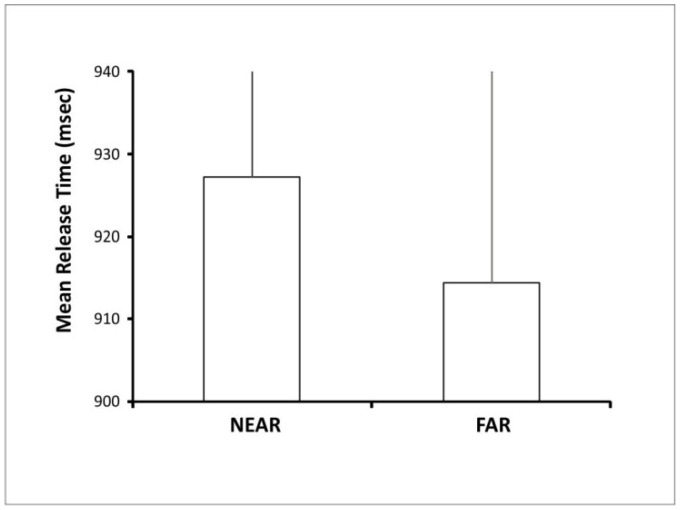
Behavioral results. The graph displays the mean release times for execution of the footstep action to far/panoramic vs. near/front views of the environmental layout. Error bars represent the standard error of the mean.

As illustrated in Figure 2, moreover, lower mean release times were observed in the far vs. the near condition and the result of a *t*-test comparison confirmed that the behavioral advantage for the footstep action in response to the far vs. near condition (far/panoramic vs. a near/restricted view) was statistically significant (t(29) = 2.4, *p* = 0.02)).

Therefore, consistently with our previous findings [7] and with the hypothesis of a preferential affordance relationship between the distant extrapersonal space and locomotion (“macro-affordance”), we found a facilitation effect for the execution of a footstep action in response to pictures of an environmental layout framed from a further extrapersonal distance.

### 3.2. EEG Event-Related Potentials

EEG analysis was focused on epochs spanning the whole prime-target interval.

To examine modulation of the ERPs by execution of the footstep action in response to pictures of an environmental layout framed from a far vs. a near extrapersonal distance, we decoded the 2 conditions starting from single-trial EEG data by means of a LDA classifier (an average 84 trials per condition were considered for the analysis). Accuracy of classification was evaluated from the onset of the prime picture (−1200 ms, 0 being the onset of target image) to approximately the onset of movement (~920 ms being the mean reaction time with respect to the onset of the target image).

**Figure 3:**
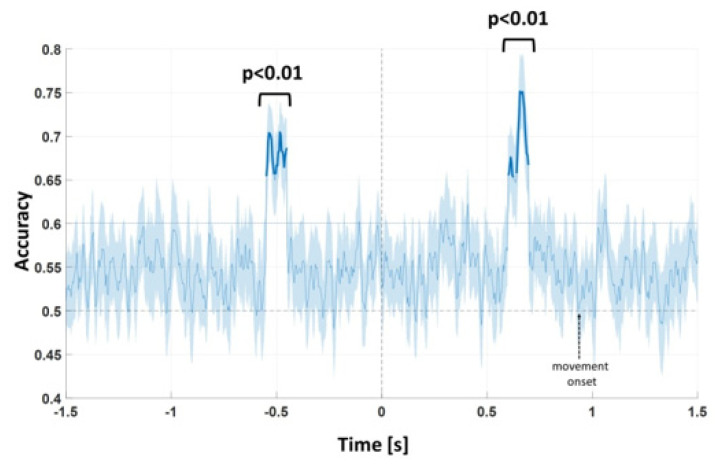
Time course of cross-validated LDA classifier (mean and standard errors across the cross-validation folds) in the time interval from −1200 to 1000 ms. Intervals in which the classifier performs higher than chance level are indicated (*p* < 0.01). Timing of the foot pedal response is indicated in the figure (~920 ms).

Figure 3 shows the time course of the cross-validated LDA classifier accuracy. Two significant intervals were found, in which the near vs. far conditions were separated (*p* < 0.01): from −550 to −450 ms and from 600 to 700 ms.

**Figure 4:**
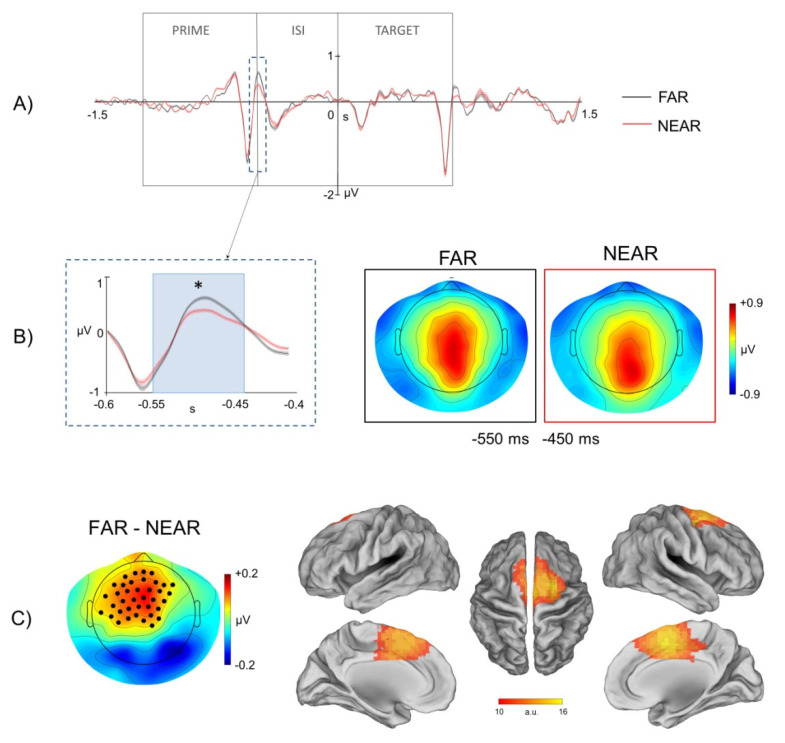
ERP modulation in the −550−450 ms interval. (**A**). Time course of the mean ERPs for the conditions of far extrapersonal distance (black) and near extrapersonal distance (red) extracted from channels over centro-frontal areas significantly different in the paired *t*-test. The time window from 1500 ms before to 1500 ms after the target image onset is shown. The time intervals of prime image presentation, ISI and target image presentation are indicated. The signals inside the dashed box are zoomed in B. (**B**). Left: Time course of the mean ERPs for the far and near condition. Vertical lines display standard error of the mean across subjects of the ERP values. Gray box indicates the time interval in which the far and near conditions were significantly different (* *p* < 0.05). Right: Mean topography across subjects of scalp electric potentials in the significant time interval (from −550 to −450 ms) for the far (black box) and near (red box) conditions. (**C**). Left: Scalp topography of the far vs. near comparison. Stars indicate EEG channels with a significant far vs. near difference, as evidenced by the paired *t*-test. Red–yellow colors indicate a positive difference, i.e., higher ERP components values in the far vs. near condition. Right: Far vs. near difference of source localization obtained using eLORETA of grand-average ERPs in the significant time interval. Yellow–red colors indicate a positive difference, i.e., higher cortical activity in the far vs. near condition.

The first time interval corresponds to the last 50 ms of the prime image presentation and the first 50 ms of the inter-stimulus interval (i.e., a window of 100 ms in between the presentation of the prime image and the ISI) (Figure 4A). As illustrated in Figure 4B, a near vs. far difference was found between EEG electrodes in medial centro-frontal areas. This difference was positive, indicating that ERP values associated with the far condition were higher than ERP values associated with the near condition. To determine the underlying cortical sources of these differences, we applied eLORETA in the significant time interval. As displayed in Figure 4C, the comparative analysis of the source localization associated with the two conditions (i.e., far vs. near) indicated a positive difference localized in regions of the medial frontal cortex evidencing a higher activation of these areas in far with respect to near conditions.

**Figure 5:**
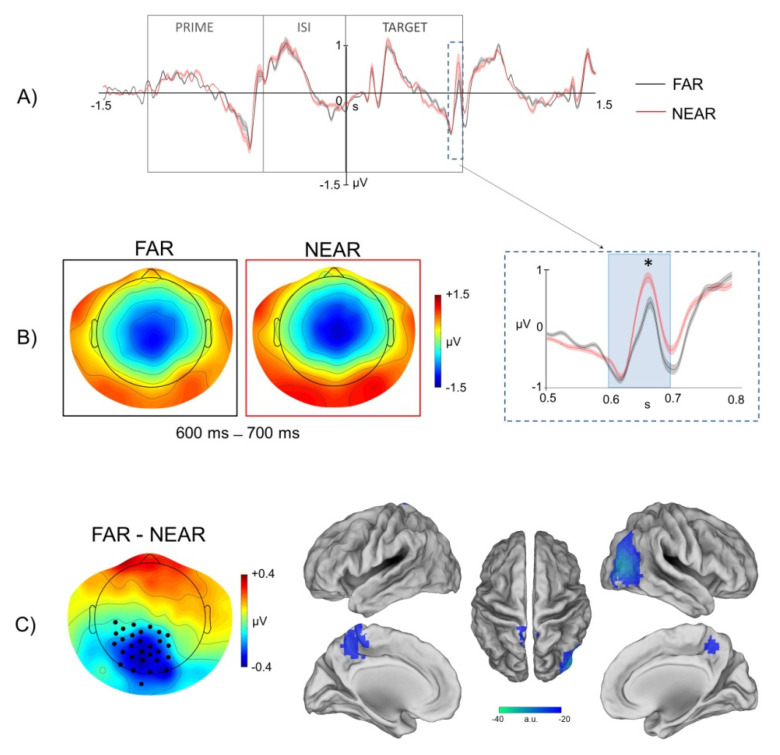
ERP modulation in the 600-700 ms interval. (**A**). Time course of the mean ERPs (black: far extrapersonal distance, red: near extrapersonal distance) of channels over posterior areas significantly different in the paired *t*-test. The time window from 1500 ms before to 1500 ms after the target image onset is shown. The time intervals of prime image presentation, ISI and target image presentation are indicated. The signals inside the dashed box are zoomed in B. (**B**). Right: Time course of the mean ERPs for the far and near condition. Vertical lines display standard error of the mean across subjects of the ERP values. Gray box indicates the time interval in which far and near conditions are significantly different (* *p* < 0.05). Right: Mean topography across subjects of scalp electric potentials in the significant time interval (from 600 to 700 ms) for the far (black box) and near (red box) conditions. (**C**). Left: Far vs. near difference of scalp topographies. Stars indicate EEG channels with a significant far vs. near difference, as evidenced by the paired *t*-test. Blue colors indicate a negative difference, i.e., the ERP components values of far condition are lower than the ERP components values of near condition. Right: Far vs. near difference of cortical source activity obtained by eLORETA of grand-average ERPs in the significant time interval. Blue colors indicate a negative difference, i.e., cortical activity lower in far than in near condition.

The second significant time interval was found from 600 to 700 ms from target onset (Figure 5A). As displayed in Figure 5B, a cluster of significant ERP modulation by framing distance of the environmental layout (far/panoramic vs. near/restricted) covered the EEG electrodes in posterior areas and was negative, indicating higher ERP values associated with the near vs. the far condition. As displayed in Figure 5C, the difference of source localization of far vs. near extrapersonal distance was also negative and was localized in bilateral regions of the anterior precuneus and in regions of the right lateral occipito-temporal cortex showing a higher activation in near with respect to far conditions.

### 3.3. Stimulus Rating

**Figure 6:**
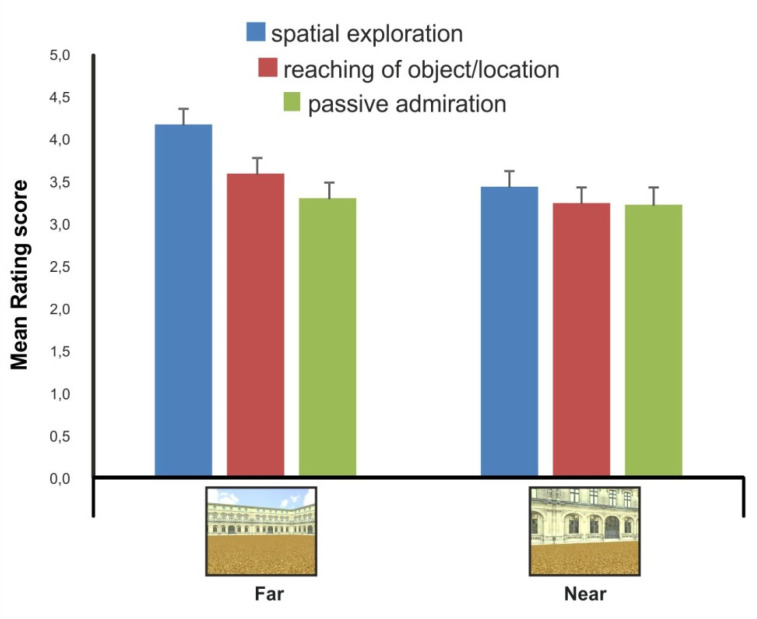
Stimulus rating (far/panoramic vs. near/front views of the environmental layout) according to the three conditions of viewer–environment interaction (spatial exploration, reaching of object/location, passive admiration). Error bars represent the standard error of the mean.

As illustrated in Figure 6, the analysis of the rating scores in the three conditions of viewer–environment interaction indicated that the passive admiration condition obtained overall lower scores than the two “active” conditions of motor interaction with the environment, i.e., reaching of object/location and spatial exploration). More relevantly, an overall higher rating scores was observed for the spatial exploration interaction condition with respect to the object/location reaching and passive admiration conditions, and this pattern was more strongly manifested in the far vs. near stimulation condition. These observations were statistically supported by the results of a within-subject repeated-measures ANOVA with distance (near, far) and condition of viewer-environment interaction (spatial exploration, reaching of object/location, passive admiration) as factors. Specifically, the ANOVA results indicated both a significant main effect of distance and condition of viewer–environment interaction and a significant two-way interaction (distance: F(1,94) = 13.2, *p* = 0.0004; affording condition: F(2,188) = 4.1, *p* = 0.01; distance by interaction condition: F(2,188) = 6.4, *p* = 0.001). Comparisons between group means using post-hoc tests (Newman–Keuls) confirmed an higher rating score for the far/exploration condition with respect to the others (all *p* < 0.05) and that the condition of viewer–environment motor interaction associated with the higher differential mean score between the far and near condition was the spatial exploration one. In other words, the spatial exploration condition (with respect to reaching of object/location & passive admiration) was the condition of viewer–environment interaction in which the greater distance effect (far > near) was observed at the level of the rating scores.

These results indicated that the 3D pictures of the far extrapersonal space preferentially evoked a viewer–environment interaction (i.e., affordance) associated with spatial exploration and navigation of the surrounding extended environment.

## 4. Discussion

The affordances of a given spatial environment (or “macro-affordances”), defined as properties of the large-scale environment providing the observer with practical opportunities of action, are essential for survival and adaptive behavior. The notion of “affordance” was originally introduced by Gibson and successively refined by other authors, including Clark, to emphasize the tight and mutual interdependence between perceptual and action-related processes [4,29].

Likewise, at the level of the neural systems, the neurophysiological model of the “affordances competition hypothesis” postulates that one fundamental principle of the brain functional organization is the elaboration of sensory information for the specification of action plans that are most appropriate for the interaction with the environment. Therefore, the role of perception for action is a central tenet of pragmatic and action-oriented views of the human information processing and brain functional architecture.

In accordance with these models, here we found that electrophysiological brain activity, recorded through scalp EEG, during visual presentation of environmental stimuli and before action planning/execution, was significantly modulated by the strength of the affordance relationship between the spatial properties of the presented environmental stimuli and the required walking-related response. In particular, we found a significant modulation of the ERP response recorded on the scalp during processing of prime images depicting the virtual environment from a far/panoramic vs. near/restricted perspective. The cortical generators associated with this modulation were localized in the dorso-medial motor cortex.

Notably, since in the current paradigm the walking-related action was executed at the onset of the target image on the basis of the perceptual match with the prime image (i.e., go when the target image is identical to the prime image), the present findings indicated a crucial contribution of the motor system in early stages of stimulus processing, even in conditions, like that of our go/no-go task, in which there was no direct mapping between the presented sensory stimulus and the required motor response. Said differently, the results of source localization of the ERPs modulations observed at the prime level as a function of extrapersonal distance, indicated an involvement of the motor system even if the presentation of the prime stimulus did not directly inform the footstep response (i.e., both go and no-go trials were included in the paradigm). In addition, it is worth noticing that no significant modulations were observed at the level of the prime stimulus presentation in occipital channels, and specifically in early visual ERP time-locked to the onset of prime images in the two conditions of environmental distance. In this respect, whereas the absence of modulations in visual ERP might be closely explained by the subtle perceptual differences between the sensory stimulation in the far vs. near conditions, the lack of observable effects suggest no differences in sensory processing of the affording properties of environmental stimuli. Notably, moreover, although the EEG inverse problem is intrinsically affected by poor spatial resolution and potential error, the source localization of prime-related ERPs modulations observed in the present study are difficult to reconcile with coarse localization errors from dorsal sensory-motor cortex to ventral visual cortex.

As for the modulations of extrapersonal distance in the prime stimulus interval, moreover, our findings showed a stronger recruitment of the motor system during perception of environmental stimuli showing a stronger affordance relationship with the walking-related action. In fact, according to our hypothesis and in agreement with our previous findings, pictures with depth cues providing the perception of further distance (e.g., linear perspective, texture gradient) were able to facilitate the execution of the subsequent footstep action (i.e., shorter footstep response times to far vs. near conditions). Importantly, moreover, such pictures of the environmental layout framed from a far vs. near extrapersonal distance also elicited a motor interaction with the environment that was preferentially associated with the process of spatial exploration and navigation.

In accordance with sensory-motor accounts of perception [30] and embodied models of predictive processing, including the “affordances competition hypothesis” outlined above [1,2,31,32], we interpret these findings as arising from a mechanism of action anticipation/prediction during perceptual processing. According to these models, indeed, the action system continually prepares multiple, parallel plans that are appropriate for the interaction with the environment and maintains these representations in working memory until one is selected for overt execution. As also shown in a recent work on architectural affordances [33], such predictive mechanism is thought to originate from the motor system and allows one to infer actions from environmental affordances, i.e., the potential to act within a specific environment.

Therefore, our findings are strikingly consistent with the definition of a mechanism of sensory-motor active inferences and perception for action [34,35,36,37] in which sensory information is elaborated for the specification of actions plans that are most appropriate for the interaction with the environment, i.e., affordances. Said differently, the finding of prime-related modulations of the ERP response originating in motor regions and reflecting the preferential affordance relationship between the presented stimuli and the required (incidental) response is interpreted in terms of embodiment of predictive processing, as if action possibilities offered by the environment could be construed as an “embodied prior belief” guiding the inference.

In addition to the far vs. near positive ERP modulation observed at the level of the prime stimulus, we also observed a target-related far vs. near negative ERP modulation. While a stronger ERP amplitude was observed during processing of prime images framing the environment from a far/panoramic vs. near/restricted view, an inversion of polarity was indeed observed during repetitions of the same images in the target-related phase of the trial. Relevantly, the source localization of this negative ERP modulation included a clearly circumscribed and specific area of activation in the dorso-medial parietal cortex closely overlapping with the anatomical definition of the foot-related sensory-motor region in the anterior precuneus [9,10,11] for which a causal role in the generation and maintenance of the “macro-affordance” effect has been demonstrated in our previous TMS study [8]. However, because presentation of the target stimulus represented the basis for the selection of the footstep response (i.e., go or no-go based on the perceptual match between target and prime), the target-related phase of the trial included both a perceptual component associated with the perceptual match of the two environmental pictures and an action-related component associated with the planning and execution of the footstep response. More specifically, since in our paradigm the presentation of the target stimulus provided the basis for executing or withholding the foot-related response and analysis of EEG signals was focused on go trials as a function of the distance condition, the ERPs modulations observed in the target-related temporal window presumably contained a mixture of perceptual and motor signals.

Notably, however, since the EEG analyses were conducted on a time window from −1200 to +1000 ms. from target onset and the average foot pedal release time was recorded at ~ 900 from the same stimulus, it is unlikely that the target-related ERP modulations observed in the interval from 600–700 following target onset were robustly affected by artefactual activity associated with footstep execution. More specifically, since the ERPs modulation by extrapersonal distance was recorded at ~200–300 ms before the onset of the footstep action and the footstep action was executed in both conditions in a time interval overlapping with the last 100 ms. of the analysis window, possible artifacts on the ERP response associated with execution of the leg movement are likely minimized in the current paradigm.

At the interpretative level, consistently with the hypothesized mechanism of action anticipation/prediction activated during prime presentation, the negative ERP modulation observed at the response (target) level could be interpreted in terms of a prediction error associated with the repetition of a perceptual condition (i.e., far) that better fulfill the expectation of guiding a locomotion-related action. Said differently, we propose that the weaker neural response to far vs. near stimuli at the target level might reflect and result from a stronger pre-activation of the locomotion-related motor program for the same stimuli at the level of the prime stimulus. Accordingly, it has been widely shown that repetition of a stimulus, as well as valid expectation that a stimulus will occur, leads to a reduction of the associated neural response, a phenomenon respectively known as repetition suppression (RS) [38] and expectation suppression (ES) [39,40,41,42]. In particular, although the RS effect is classically attributed to neuronal adaptation, as a result of a bottom-up modulation of neuronal sensitivity to a stimulus, RS can also arise from top-down predictive mechanisms, as a comparison between top-down activity (reflecting stimulus expectation) and bottom-up activity (reflecting the currently available or presented stimulus) [43,44]. From this perspective, the neural activity attenuation in response to stimulus repetition would reflect a relative reduction in top-down “prediction error” associated with processing of expected compared to unexpected sensory stimuli [39,40,41,42].

On this basis, here we propose that the “macro-affordance” effect observed in the present EEG study both at the level of the behavioral and the neural response was subserved by a corresponding mechanism of top-down prediction and consequent prediction error. We therefore advocate the hypothesis that, similarly to learnt statistical regularities in the environment (i.e., repetition likelihood), the affordance relationship between the spatial properties of environmental stimuli and the locomotion action might be orchestrated by a neurophysiological mechanism of expectation and prediction error. Indeed, if one starts from the assumption, based on the behavioral data, that a more accurate or stronger prediction for locomotion is generated during processing of prime stimuli from the far vs. the near condition, then it might be predicted that repetition of the far affording condition in the target phase of the trial (in which the footstep response was executed) could be associated with a reduction of the prediction error with respect to the near condition. Therefore, here we propose that the behavioral facilitation effect observed during execution of a footstep action in response to repeated presentation of pictures framed from a far vs. near perspective is supported by a neurophysiological mechanism of predictive coding activated by the affordance relationship between the far perceptual stimulus and the required response during the first stimulus presentation (i.e., prime) and reflected in a smaller prediction error during stimulus repetitions (i.e., target).

Accordingly, the ERP modulation observed in our data in the time interval from 600 to 700 ms post-target was consistent with RS modulation of event-related neurophysiological signals by repetition probability previously observed in relatively late time windows (>300 ms) post-stimulus [40,42]. In this respect, it has been proposed that RS and ES effects may represent a manifestation of prediction error at different time scales, with repetition suppression occurring earlier, as a consequence of local transition probabilities, and suppression by expectation occurring later, as a consequence of higher-order expectations, based on more complex statistical regularities within the environment [43,45].

Future developments of the current research will surely include additional follow-up analysis of the current EEG data, for example, by focusing on modulation of the EEG rhythmic activity in frequency bands, like mu or beta, that are traditionally thought to reflect the activity of sensory-motor areas [46]. In addition, future directions will possibly include the contextualization and generalization of the macro-affordance effect within the broader theoretical framework of the affordance and embodied cognition theory as well as the understanding of its impact on clinical and experimental research in the field of aging and movement-related disorders. For example, an interesting possible application of the current findings is the emerging research field of embodied cognition in aging [47] for the development of rehabilitation protocols for boosting or restoring walking-related locomotion based on neurobehavioral sensory-motor facilitation.

## 5. Conclusions

In summary, our EEG recordings suggests that RS/ES effects might be also observed in sensory-motor regions of the dorso-medial fronto-parietal cortex and that these effects might be modulated by the affording properties of repeated perceptual stimuli (i.e., environmental images) during an incidental priming paradigm in which the relevant dimension (i.e., spatial distance) was implicitly processed. This observation closely supports the findings of RS effects in sensory regions even in absence of perceptual awareness [48] and extends the observation of implicit mechanisms of predictive processing and prediction error to key cortical nodes of the parieto-medial temporal pathways for spatial cognition and navigation [14]. At a more speculative level, our findings concur to support and provide empirical evidence to the evolutionary stance advocated by Friston and Allen that “the nervous system is itself selected by evolution to minimize prediction error within a particular ecological niche and that evolution is itself a predictive processing engine” [49].

## Data Availability

The data presented in this study are available on request from the corresponding author since study participants were assured that raw data would remain confidential and would not be shared.
